# Diffusion and Binding of Mismatch Repair Protein, MSH2, in Breast Cancer Cells at Different Stages of Neoplastic Transformation

**DOI:** 10.1371/journal.pone.0170414

**Published:** 2017-01-26

**Authors:** Justin Sigley, John Jarzen, Karin Scarpinato, Martin Guthold, Tracey Pu, Daniel Nelli, Josiah Low, Keith Bonin

**Affiliations:** 1 Department of Physics, Wake Forest University, Winston-Salem, North Carolina, United States of America; 2 Department of Microbiology and Immunology, Medical University of South Carolina, Charleston, South Carolina, United States of America; 3 OVPR, University of Miami, Miami, Florida, United States of America; Pennsylvania State Hershey College of Medicine, UNITED STATES

## Abstract

The interior of cells is a highly complex medium, containing numerous organelles, a matrix of different fibers and a viscous, aqueous fluid of proteins and small molecules. The interior of cells is also a highly dynamic medium, in which many components move, either by active transport or passive diffusion. The mobility and localization of proteins inside cells can provide important insights into protein function and also general cellular properties, such as viscosity. Neoplastic transformation affects numerous cellular properties, and our goal was to investigate the diffusional and binding behavior of the important mismatch repair (MMR) protein MSH2 in live human cells at various stages of neoplastic transformation. Toward this end, noncancerous, immortal, tumorigenic, and metastatic mammary epithelial cells were transfected with EGFP and EGFP-tagged MSH2. MSH2 forms two MMR proteins (MutSα and MutSβ) and we assume MSH2 is in the complex MutSα, though our results are similar in either case. Unlike the MutS complexes that bind to nuclear DNA, EGFP diffuses freely. EGFP and MutSα-EGFP diffusion coefficients were determined in the cytoplasm and nucleus of each cell type using fluorescence recovery after photobleaching. Diffusion coefficients were 14–24 μm^2^/s for EGFP and 3–7 μm^2^/s for MutSα-EGFP. EGFP diffusion increased in going from noncancerous to immortal cells, indicating a decrease in viscosity, with smaller changes in subsequent stages. MutSα produces an *effective* diffusion coefficient that, coupled with the free EGFP diffusion measurements, can be used to extract a pure diffusion coefficient and a pseudo-equilibrium constant K*. The MutSα nuclear K* increased sixfold in the first stage of cancer and then decreased in the more advanced stages. The ratio of nuclear to cytoplasmic K*for MutSα increased almost two orders of magnitude in going from noncancerous to immortal cells, suggesting that this quantity may be a sensitive metric for recognizing the onset of cancer.

## Introduction

Advances in biophysics are providing opportunities to establish connections between cell mechanics and cellular functions. Cancer research is one area where this is especially true. Recent reports indicate that cancerous and non-cancerous cells have different physical and mechanical properties arising from biochemical alterations as normal cells transform to cancerous cells [1,2]. There is, however, significant ambiguity about how these properties change. One source of ambiguity lies with the type of cell being studied. For example, human bladder cells [3], mammary epithelial cells [4–6], pancreatic epithelial cells [7,8], and mouse fibroblast cells [9] have shown increased deformability in cancerous cells compared to normal cells; in contrast human hepatocytes [10], myeloid and lymphoid leukemia cells [11], and Lewis lung carcinoma mouse cells [12] have shown decreased deformability with cancer. The mechanical properties of tissues may differ from the properties of individual cells, because tissue also contains the extracellular matrix. It has also been shown that the surrounding matrix can drive cell physical development [13]. Stiffness maps from benign human breast biopsies show uniform stiffness profiles characterized by a single peak, in contrast to malignant tissues which have broader peaks resulting from tissue heterogeneity and a characteristic low-stiffness peak representative of cancer cells [14]. Another reason for different results is the method being used to measure the mechanical properties of cells. Atomic force microscopy has been used to test local membrane properties [11,15], while micropipette aspiration [7,16] and microplate stretchers [17] measure global cell properties.

In addition, for a given cell type, there could be differences in mechanical properties as determined from the inside versus outside the cell, based on which cellular components are important in the two different zones. For example, mechanical properties measured from outside the cell via the methods mentioned in the last paragraph are most likely strongly determined by both the actin cortex that resides as a thin layer just beneath the cellular membrane as well as by cytoskeletal structural components such as actin filaments, microtubules, intermediate filaments, and cytoskeletal septins [18]. In fact recent work on the cortex has been carried out using Atomic Force Microscopy to try and explain the systematically higher stiffness values measured with sharp AFM tips compared to larger tips [19]. A recent report examines the coupling between the outer membrane and the actin cortex [20].

Here, we report diffusion measurements of the mismatch-repair protein MSH2 inside cells using fluorescence recovery after photobleaching (FRAP). FRAP is a well-established method for measuring protein mobility in live cells [21]. Although there has been some evidence that the high powered bleaching pulse used in FRAP may alter the diffusion-dependent kinetics in a cell by altering local viscosity [22], it has been shown that the FRAP method is quite robust, giving solid and consistent diffusion results if properly applied and analyzed [23]. Other techniques have been developed to probe the intracellular environment. Particle tracking provides a very localized measurement of cellular viscosity without having to take into account the contribution of the plasma membrane [6,24]. A relatively new method called raster image correlation spectroscopy (RICS) can accurately measure molecular diffusion in the microseconds-to-seconds timescale [25] in living cells using a laser scanning confocal microscope. In relation to cancer, this technique has been used to measure the diffusion of P53 in live HeLa cells treated with DNA damaging agents [26]. However, a disadvantage of RICS is that the active fluorophore concentration must be less than ~ 80 molecules in a focal volume [27]. Since it is difficult to control expression in cells, this condition is frequently violated.

Genetic instability is the underlying driver of the neoplastic process, playing an important role in both initiation and progression of cancers [28,29]. As such, DNA repair processes play a central role in the prevention of cancer by correcting damage caused by exogenous and endogenous sources. The mismatch repair process corrects base substitution mismatches and insertion-deletion mismatches resulting from replication errors and recombination events. The first step in the mismatch repair process is recognition of the mismatch. This is performed by one of two heterodimeric protein complexes consisting of MutS homologous proteins MSH2 and MSH6 (forming MutSα), or MSH2 and MSH3 (forming MutSβ). These proteins must find their way to the DNA within the nucleus and be able to selectively bind to their target, which involves specific conformational changes [30].

In the current study, we use FRAP to measure the diffusion of MSH2-EGFP constructs in human mammary epithelial cells engineered to mimic four different stages of neoplastic transformation. Here, we label these four stages noncancerous, immortal, tumorigenic, and metastatic. Note that there is no universal model for stages that cancer cells traverse on the way from normal to metastatic, but there is strong evidence for at least three mutational stages [31], and up to six stages that have been delineated for colon cancer cells [31]. Thus, we are using our cells, which embody different levels of mutational compromise, as an approximate model for neoplastic transformation.

Finally, to discuss diffusion and binding of MSH2 in cells, we need to briefly consider the most likely form that MSH2 takes in cells. There is good evidence that MSH2 and MSH6 bind to form the heterodimer MutSα in the cytoplasm before entering the nucleus [32]. This group found that MSH6 deficient cells lacked MSH2 in the nucleus. Additional data of this group, which separately analyzed cytoplasmic and nuclear extracts of both MSH2 and MSH6, showed that all the MSH2 was bound to MSH6 in both regions. Modrich [33] also reported that in mammalian cells, MutSα comprises 80–90% of cellular MSH2. Edelbrock, et al.[34] provide an extensive and nuanced review of MSH2 complexes and behavior in cells, and they conclude that the hMSH2 not bound with hMSH6 is most likely in the form of hMutSβ. This is likely since MSH2 does not have any nuclear localization sequences (NLS), and both MSH6 and MSH3 do have NLS sequences. However, it has also been observed that MSH2 can be imported from the cytoplasm into the nucleus via the importinβ/α3 protein dimer [35], and importinβ has an NLS. There is also evidence that MSH2 binds to other proteins, e.g. MAX, a heterodimeric partner of c-MYC, a transcription factor that regulates the cell cycle [36], and e.g. to a very large complex called BASC, that complexes BRCA1, a human tumor suppressor protein with MSH2, MSH6, and other proteins [37]. Since the basic results scale with protein mass according to M^1/3^, this means that only very large differences in protein masses will result in significant differences for the diffusion coefficients. For example, a factor of 8 increase in mass would only produce a factor of 2 decrease in diffusion. This result, coupled with the fact that the extensive literature on MSH2 is mostly focused on its behavior as a member of the MutSα complex, causes us to hereafter treat our EGFP-labelled MSH2 as an EGFP-labelled MutSα complex. We will hereafter denote the MSH2 complex we are measuring as MutSα-EGFP.

## Materials and Methods

### Cell Culture and Transfection

For the current study, we use 1) human mammary epithelial (HME) cells and 2) three cancer cell lines. The two non-metastatic cancer cell lines were engineered by the Weinberg laboratory to simulate progressive stages of neoplastic transformation [38]. These cells include low-passage normal HME cells (Lonza), HME cells expressing hTERT and large T antigen (LT) (HME +LT, TERT), and HME cells expressing hTERT, LT, and H-rasV12 (HMLER). The expression of hTERT lengthens and maintains telomeres, allowing previously senescent cells to exceed the Hayflick limit. Increased telomerase activity alone was found to be sufficient to immortalize post-M0 HME cells but not pre-M0 HMEs [39]. Expression of hTERT and deactivation of the pRB and p53 pathways by LT were sufficient to create immortal HMECs that did not form subcutaneous tumors in mice [38]. HMECs expressing hTERT, LT, and H-rasV12 were shown to form tumors in nude mice with 52% efficiency [38]. Finally, we also included the human metastatic breast cancer cell line MDA-MB-231; these cells were obtained from ATCC (MDA-MB-231 ATCC HTB-26), and are derived from a pleural effusion from a patient. These metastatic cells express the WNT7B oncogene [40] and in both antilymphocyte serum treated BALB/c mice and nude mice these cells form poorly differentiated grade III adenocarcinomas. A summary of the cell types is listed in Table 1.

**Table 1:** The human mammary epithelial cell types used here.

Cell Type	Immortal	Tumorigenic	Metastatic	Label*
HME	no	no	no	noncancerous
HME + LT, hTERT	yes	no	no	immortal
HMLER	yes	yes	no	tumorigenic
MDA-MB-231	yes	yes	yes	metastatic

* These labels are used throughout the paper.

Cells were cultured in 35 mm poly-d-lysine coated glass bottom dishes (MatTek) at 37°C in 5% CO_2_ in Mammary Epithelial Growth Medium (MEGM, Lonza) with bovine pituitary extract (BPE) at a final concentration of 52 μg/ml. Cells were passaged at about 70–90% confluence. Culture medium was replaced every 2 days. For fluorescence imaging, cells were transiently transfected with EGFP and MSH2-EGFP plasmids using Lipofectamine 2000 (Life Technologies). For each 35 mm dish, 200 μl of Opti-MEM reduced serum media (Life Technologies), 10 μl Lipofectamine LTX, 2.5 μl Plus Reagent, and 2.5 μg of DNA plasmid were added. Cells were transfected at 50–80% confluence and imaged within 1–3 days after transfection.

Cell lines (all human mammary epithelial cells) were obtained from the following sources: noncancerous cells—Lonza HMEC–Human Mammary Epithelial Cells (Catalog #: CC-2551, Walkersville, MD, orders placed June 2011, August 26, 2013 –Lot # 0000220519. The immortal and tumorigenic non-metastatic human mammary epithelial cells developed in the Weinberg laboratory [38] were provided by Karin Scarpinato in January 2012. Metastatic human mammary epithelial (MDA-MB-231) cells were obtained from the American Type Culture Collection (ATCC, Manassas, VA, Catalog #: ATCC^®^ HTB-26, and purchased on March 25, 2013, Lot# 599228346). They were also purchased from the ATCC in August 2013 under Catalog#: ATCC HTB-26.

### Microscopy

Fluorescence imaging was done on a Zeiss LSM 710 laser scanning confocal microscope mounted on an AxioObserver Z.1 inverted microscope using a Plan-Apochromat 63x/1.4 NA oil-immersion objective. Cell dishes were placed in an enclosed Pecon PS1 incubation system to maintain the local environment at 37°C with 5% CO_2_ and high humidity to prevent media evaporation while imaging.

**Fig 1:**
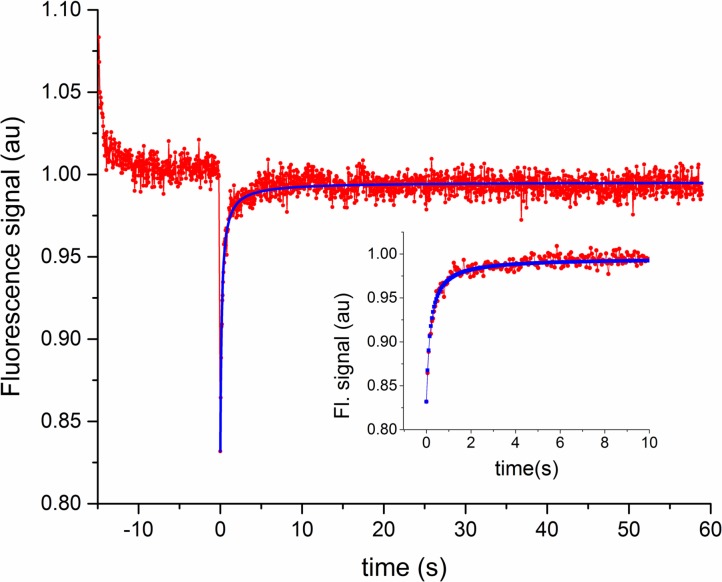
FRAP fluorescence vs. time. Representative FRAP curve showing the recovery of fluorescence with time after the photobleach, which ended at t = 0. Red points/line are the experimental data, and the blue points/line are the best fit to the data using Eq (2). These data are from a noncancerous cell in the cytoplasm. Inset: Same data and fit shown for the first 10 seconds to better show the details during recovery. For the full curve, note that only the 1200 points starting at t = 0 and after were corrected for photobleaching in order to perform the FRAP fit. Thus the first 300 points before the bleach at t = 0 exhibit photobleaching.

The FRAP photobleach geometry consisted of a disk of diameter equal to 36 pixels with the 488 nm laser set to 100% power, which corresponded to a measured power at the sample of 330 μW, and an irradiance of 2.5 kW/mm^2^ (Thorlabs Optical Power Meter model PMD100; readout head model S170C). The photobleach scan consisted of a single bleach cycle occurring after the first 300 scan cycles. Pixel size was set to 0.088 μm and the pixel dwell time was 12.5 μs during the photobleaching scan. For FRAP imaging, cycles of 1500 frames were collected for each run with a scan area of 512x50 pixels. We took 300 prebleach scans due to the significant observational photobleaching present during the first 10 seconds of imaging as seen in Fig 1. Pixel size was set to 0.088 μm and the pixel dwell time was set to 1.58 μs. The 488 nm laser power during image scanning was 1% of full power. For each cell, FRAP data was gathered once in the cytoplasm and once in the nucleus. Images were collected for all four cell types. Cells undergoing mitosis and rounded cells (indicating potential cell death) were excluded from the study. For each run all 1500 images were saved as well as a text file containing the FRAP intensity vs. time results, as calculated by the Zeiss Zen software. The FRAP intensity at each time point consisted of the average intensity in the photobleach region located in the center of the image. A minute after the end of a 1500 image scan, a second 1500 image scan was taken without the photobleach after cycle 300. This second image scan was used to establish the bleaching that occurs due to photoimaging. The photoimaging data were fit with a decaying exponential, and the resulting curve was used to correct for the effects of bleaching due to photoimaging. This was done by dividing the exponential fit curve into the FRAP data, as recommended by Mueller, et al. [23].

### FRAP Theory and Analysis

A representative FRAP fluorescence signal vs. time data set, with fit, is shown in Fig 1. Also shown is the pre-bleach part of the data. The fluorescence signal was in the wavelength range of 493–650 nm, and is the average signal from a well-defined region of interest that was within the photobleached region, and was detected by a photomultiplier tube in the Zeiss LSM 710 confocal microscope. The postbleach data have been corrected for bleaching caused by photoimaging. Several steps were involved in extracting a diffusion coefficient, *D*, from the fitted curve. Two key measured parameters are needed to determine *D*, given by

$$D=\frac{r_{e}^{2}}{4\tau_{D}},$$ (1)

where the recovery or diffusion time, τ_*D*_, is found by fitting a curve (Eq 2, below) to the FRAP data, and where the effective bleaching beam radius *r*_*e*_ is obtained by a careful analysis of the bleaching spot pattern in the first frame after the photobleach (Eq 5, below). This analysis was found to have two advantages over other FRAP analysis approaches: 1) it accounts for the diffusion that occurs during the photobleach and 2) the method for determining *r*_*e*_ in Eq 1 accounts for the bleach spot size up to a certain size limit (for details see Refs [41,42]). In an attempt to more accurately account for the diffusion that occurs during bleaching, Braga, et al. [43] developed a FRAP approach, in which the bleaching beam profile in the first image after the photobleach was used to determine a more accurate value for the diffusion coefficient. McNally also recognized the importance of understanding bleaching profiles [23]. McNally and his group developed a model that consisted of breaking up the profile into two regions: a saturated inner region, and an outer region with a characteristic Gaussian profile, which resulted in accurate analysis of FRAP data. Following the work of Braga, et al. [43], Kang et al. [41,42] developed simpler expressions for characterizing the FRAP fluorescence signal versus time using the beam profile, as well as more deeply investigated the method in a wide variety of cells and in a set of EGFP controls. In the following, we summarize our use of the Kang expressions and approach to analyze our FRAP data.

Note that our data could be fit, with good reduced chi-squared values, with a model that only took into account diffusion, ignoring additional, explicit binding terms. A different model would be needed to simultaneously account for diffusion and binding together—for a discussion of FRAP models with explicit parameters to fit both diffusion and binding, see [23]. The use of a simple diffusion model does not mean that binding is absent, but that a model that explicitly requires terms that directly rely on binding parameters, such as association and dissociation rates, is not needed to adequately fit the data. In some cases, e.g. for our MSH2-EGFP data presented and analyzed in this paper, an *effective* diffusion coefficient is obtained from the FRAP model, and this term depends on the free diffusion and binding parameters. In the case of an effective diffusion coefficient additional measurements are used to extract binding information from the effective diffusion coefficient. Typically, what is done is that the diffusion of a protein known to remain free is measured and used to estimate the free diffusion of the complex that gave rise to an effective diffusion. Further details of this approach are given in the Results section.

The FRAP fluorescence signal as a function of time, *F(t)*, was normalized to its prebleach value (also correcting for photobleaching during imaging as discussed above), using a model with the form [41]

$$F(t)=F_{i}\left[1-\frac{K}{1+\gamma^{2}+\frac{2t}{\tau_{D}}}\right]M_{f}+(1-M_{f})F_{0}.$$ (2)

Here *F*_*i*_ is the initial, prebleach fluorescence signal, *F*_*0*_ is the initial postbleach fluorescent signal, γ is the ratio of the nominal and effective bleach profile radius *(γ* *= r*_*n*_*/r*_*e*_), and *M*_*f*_ is the mobile fraction, given by the relation

$$M_{f}=\frac{F_{\infty }-F_{0}}{F_{i}-F_{0}}.$$ (3)

Here the quantity *F*_∞_ is the fluorescent signal in the FRAP "plateau" region, i.e. at long times after the recovery (*t* → ∞). Finally the quantity K is the bleaching depth parameter, which physically represents how deeply the fluorophores are bleached by the high irradiance laser during the bleaching scan. This parameter must be determined prior to fitting the FRAP data with Eq (2). The bleach depth *K* is found by fitting the first postbleach image bleaching profile with the following function

$$C(x,y,0)=C_{i}\left[1-K\exp\left(-\frac{2(x^{2}+y^{2})}{r_{e}^{2}}\right)\right],$$ (4)

where *r*_*e*_ is the effective bleach radius, and the concentration of fluorophores at position (*x*,*y*) at time *t = 0* is *C*(*x*,*y*,0). It has been found that Eq (4) is a good model for the postbleach profile [41].

To carry out our analysis we first average all the cytoplasm postbleach profile data for a given session and cell type, and then we separately average all the nuclear data for the same session and cell type. Matlab programs were used to normalize the FRAP signals to their prebleach values, and a script file was used to fit the function

$$f_{norm}(r)=1-K\exp\left(-\frac{{\left(x-x_{c}\right)}^{2}}{r_{ex}^{2}}-\frac{{\left(y-y_{c}\right)}^{2}}{r_{ey}^{2}}\right),$$ (5)

where *r*_*ex*_ and *r*_*ey*_ are separate photobleach beam profile radii along the x and y axes, and *x*_*c*_ and *y*_*c*_ are the center positions of the fluorescence bleaching distribution along the same axes. We determine the equivalent radius, *r*_*e*_, by taking the value along the axis with the most data (we record 512 pixels of data along the x-axis and only 50 along y), which means setting *r*_*e =*_ *r*_*ex*_. One final note about the fitting–early points were weighted more heavily than points in the plateau region by the logarithmic weighting as suggested by Mueller, et al.[23]

### FRAP Controls

Important control experiments demonstrating the validity of the FRAP approach to determine D, as described in the previous section, were carried out by Kang et al. [41,42]. In these experiments, the diffusion coefficients of EGFP in 40%, 50%, and 70% solutions of glycerol (w/v) were determined and checked against the value determined by the Stokes-Sutherland-Einstein relation

$$D=\frac{k_{B}T}{6\pi \eta r_{h}},$$ (6)

where T is the temperature, *k*_*B*_ = 1.38 x 10^−23^ J/K is the Boltzmann constant, η is the viscosity and *r*_*h*_ is the hydrodynamic radius of the particle. We carried out similar control experiments using the same solutions of three different fluid viscosities, η. We, furthermore, compared our D values to a rescaled, accurate literature value [44]. The most accurate reported experimental value that we could locate in the literature [44], based on its quoted error, for the EGFP diffusion coefficient in water (100 mM phosphate-citrate buffer, pH 7.5) at room temperature (22°C) is *D*_*Petrasek*_ = 95 +/- 2 μm^2^/s. Using the viscosity of water at 22.5°C, η_*w*_ = 0.943 mPa·s, and the viscosities for our glycerol-water mixtures at temperature T, η_*glyc-w*_ (T), from the temperature-dependent model of Cheng [45] and glycerol-water data for η_*glyc-w*_ (T) from Segur and Oberstar [46], we determined D values for the glycerol mixtures using

$$D_{\exp}=D_{Petrasek}\left(\frac{\eta_{w}(22{}^{\circ}C)}{\eta_{glyc-w}(T)}\right)\times \left(\frac{T}{295}\right).$$ (7)

Here 295 K is the temperature at which the measurements were carried out by Petrasek, at al. [44]. The aqueous component of our glycerol solutions contained 50 mM TRIS, pH 8.3 to assure EGFP activity. Averaged measurements of EGFP diffusion in the three control solutions using our FRAP method are summarized in Table 2, along with expected values from Eq (7).

**Table 2:** EGFP diffusion measurements in water-glycerol mixtures.

% glycerol (w/v) in buffer	D_EGFP_ (μm^2^/s)—this work^a^	D_exp_(μm^2^/s)^b^
40	30 +/- 2 (33)	29 +/- 1.3
50	20 +/- 1.1 (11)	17.3 +/- 0.7
70	4.9 +/- 1.4 (13)	5.0 +/- 0.4

^a^FRAP measurements, values are averages +/- standard errors in the mean (SEM); numbers in parentheses correspond to the number of measurements.

^b^Scaled from an accurate value measured at 22.5°C in water, using Eq 7.

Table 2 shows good agreement between all measured values for the control solutions and the expected values scaled from the measurement by Petrasek et al. [44].

### Control Measurement for Protein Mobility across Nuclear Membrane

We performed fluorescence loss in photobleaching (FLIP) experiments to determine if the EGFP and MutSα-EGFP constructs were able to freely diffuse through the cell and cross the nuclear membrane. In these experiments, the entire nucleus of a cell was repeatedly bleached while monitoring the fluorescence intensity of a region of interest in the cytoplasm. The fluorescence intensity in the cytoplasm continuously decreased as the fluorescent proteins diffused into the nucleus and were bleached. When the bleaching pulse was turned off, the fluorescence intensity in the nucleus quickly recovered indicating no significant barrier to EGFP or MutSα-EGFP transport into the nucleus.

## Results

**Fig 2:**
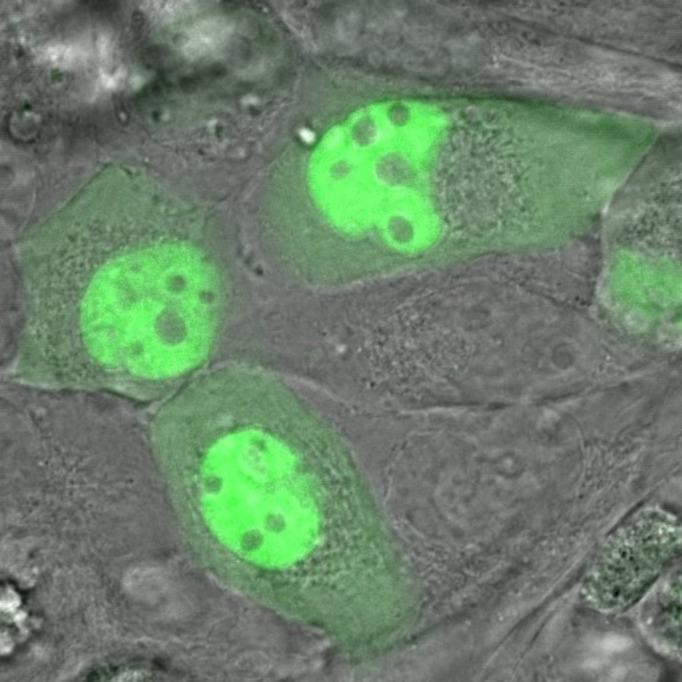
Fluorescent & DIC images of cells. Two combined images–a differential interference contrast image (grayscale) with a MutSα-EGFP image overlayed in green. Note the significant increase in MSH2 concentrations in the nuclei. Inhomogeneities are also readily observed in both cell regions. In particular we note, that based on a quantitative analysis of this image using ImageJ, the nucleoli have about half the MSH2-EGFP concentration of the surrounding nucleus. These are immortal cells.

We performed FRAP diffusion measurements on EGFP and MutSα-EGFP in two different cellular regions: the cytoplasm and the nucleus, and on four different cells: noncancerous, immortal, tumorigenic, and metastatic, representing four different stages of neoplastic transformation. A sample image of the fluorescence of MutSα-EGFP for immortal cells is given in Fig 2.

Table 3 summarizes the results for all 16 different combinations of experimental conditions (2 proteins, 2 cell regions, 4 cell types).

**Table 3:** Diffusion coefficients of human mammary epithelial cells for EGFP and MutSα-EGFP in cytoplasm and nucleus using FRAP.

	EGFP		MutSα-EGFP	
*Cell Type*	*Cytoplasm*	*Nucleus*	*Cytoplasm*	*Nucleus*
*noncancerous*	24 +/- 1.2 (55)	16.7 +/- 0.74 (54)	3.5 +/- 0.2 (29)	5.8 +/- 0.4 (27)
*immortal*	14 +/- 1.0 (34)	18 +/- 1.3 (33)	7.5 +/- 0.5 (18)	2.9 +/- 0.12 (33)
*tumorigenic*	21 +/- 1.6 (33)	20 +/- 1.1 (33)	2.5 +/- 0.3 (20)	3.5 +/- 0.4 (18)
*metastatic*	22 +/- 1.9 (25)	22 +/- 1.9 (25)	4.75 +/- 0.4 (28)	5.1 +/- 0.3 (28)
average*	19.0 +/- 0.7 (147)	18.1 +/- 0.5 (145)	3.6 +/- 0.14 (95)	3.4 +/- 0.10 (106)

The table contains mean values +/- standard errors of the means. The number of independent cells measured that contributed to each average is given in parentheses.

*This row consists of weighted averages +/- SEM values. The weights w_i_ are w_i_ = 1/σ_i_^2^, where σ_i_ is the standard error of the mean for the *i*-th measurement [47].

**Fig 3:**
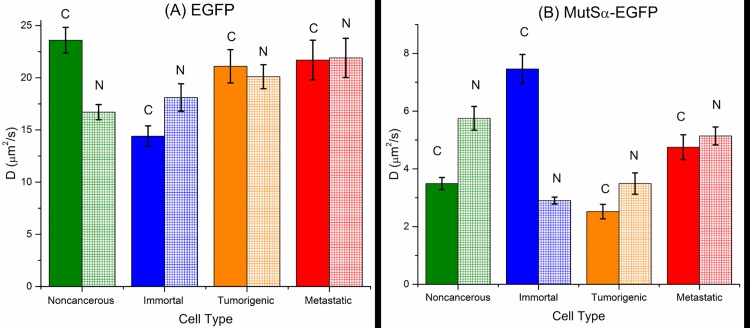
Diffusion data. Diffusion data separated by protein, cell type and cellular region: (A) EGFP and (B) MutSα-EGFP. Each x-axis is ordered according to the neoplastic progression of the cancer cell–noncancerous on the left and metastatic on the right. For each cell type cytoplasmic and nuclear values are plotted next to each other (cytoplasm–solid; nucleus–hatched). Error bars are +/- SEM.

In Fig 3A & 3B we plot the diffusion data organized by protein.

We also present the results of analyzing the t-statistic using Student's t-test on all possible pairs in the data set. To make the data more readable, it was separated into 2 different tables, based on protein type. Differences that are statistically significant (p < 0.05) have asterisks whose number indicates the level of statistical significance–see Tables 4, 5 and 6. Differences that are not statistically significant (p > 0.05) are labeled with the symbol ns.

**Table 4:** Symbols used to indicate statistical significance in associated paired t-test tables.

Symbol	Meaning
ns	p > 0.05
*	p < 0.05
**	p < 0.01
***	p < 0.001

**Table 5:** Statistical significance of results for the *EGFP proteins*.

Cell		*noncancerous*		*immortal*		*tumorigenic*		*metastatic*	
	region	*cyto*	*nuc*	*cyto*	*nuc*	*cyto*	*nuc*	*cyto*	*nuc*
noncan	*cyto*		***	***	**	ns	*	ns	ns
	*nuc*			ns	ns	*	*	*	*
immort	*cyto*				*	***	***	**	***
	*nuc*					ns	ns	ns	ns
tumor	*cyto*						ns	ns	ns
	*nuc*							ns	ns
meta	*cyto*								ns
	*nuc*								

**Table 6:** Statistical significance of results for the *MutSα-EGFP proteins*.

Cell		noncancerous		immortal		tumorigenic		metastatic	
	region	*cyto*	*nuc*	*cyto*	*nuc*	*cyto*	*nuc*	*cyto*	*nuc*
noncan	*cyto*		***	***	*	**	ns	**	***
	*nuc*			*	***	***	***	ns	ns
immort	*cyto*				***	***	***	***	***
	*nuc*					ns	ns	***	***
tumor	*cyto*						*	***	***
	*nuc*							*	**
meta	*cyto*								ns
	*nuc*								

As McNally and others have pointed out in papers describing the interpretation of FRAP results [48,49], when a simple diffusion model works well on a species, such as the MutSα protein, that is known to physically bind in the sample to other objects (such as DNA in the nucleus or other proteins in the cytoplasm or nucleus), then one can extract some binding information from the measured diffusion coefficients. In these cases the diffusion coefficient is called an effective diffusion coefficient, *D*_*eff*_, that is related to the diffusion coefficient in the absence of binding, *D*_*f*_, by [48,49]

$$D_{eff}=\frac{D_{f}}{\left(1+(k_{on}^{*}/k_{off})\right)},$$ (8)

where the pseudo-equilibrium constant $K*=k_{on}^{*}/k_{off}$, is the ratio of the pseudo-on rate and the off rate of the binding. Physically, for binding to a nearly fixed partner, the pseudo-equilibrium constant is the ratio of bound/free molecules–see [48] for details. In fact, the interpretation of the pseudo-equilibrium constant can be somewhat complex, depending on the physical situation, since this model cannot distinguish whether there are one, two, or more binding states that are at play in the binding interaction. In cases where multiple binding states are at play then the deduced ratio $k_{on}^{*}/k_{off}=K*$ is actually the average of the ratios for the different states [50]. The free diffusion coefficient *D*_*f*_ is to be calculated [48] by scaling the diffusion coefficient measured for a species known to not bind, i.e. freely diffuse, such as EGFP. Scaling is performed using the standard relationship [48,49]

$$D_{f}=D_{free-MutS\alpha -EGFP}={\left(\frac{M_{EGFP}}{M_{MutS\alpha -EGFP}}\right)}^{1/3}D_{EGFP},$$ (9)

where *M*_*EGFP*_ = 26.9 kDa is the mass of an EGFP protein, and *M*_*MutSα—EGFP*_ = 257.7 kDa + 26.9 kDa = 285 kDa is the mass of the MutSα-EGFP fusion protein. We note that the mass of the MutSβ-EGFP fusion protein (259 kDa) and the mass of importinβ/α3-EGFP (286 kDa) are very close to that of MutSα-EGFP.

As stated in the Abstract and Introduction, we are assuming that MSH2 is in the form of the MutSα complex with *M*_*MutSα-EGFP*_ = 285 kDa. Using this mass we can generate estimates for *D*_*f*_ in Eq 9 by using the free EGFP diffusion coefficients and the masses of the free EGFP and the MutSα-EGFP complex. With values for *D*_*f*_ we can then determine the pseudo-equilibrium constants K* by using the following expression that can be obtained from Eq 8

$$K*=\frac{k_{on}^{*}}{k_{off}}=\frac{D_{f}}{D_{eff}}-1.$$ (10)

Thus we can use the *D*_*f*_ values (and the values for *D*_*eff*_ in Table 3) in Eq 10 to obtain values for the pseudo-equilibrium constant $K*=k_{on}^{*}/k_{off}$. These results are summarized in Table 7.

**Table 7:** Free diffusion coefficient estimates assigned to the MutSα-EGFP complex and values for the pseudo-equilibrium constants $K*=k_{on}^{*}/k_{off}$ in the different cell types and regions.

	*D*_*f*_ (μm^2^/s)		$K*=k_{on}^{*}/k_{off}$	
*Cell Type*	Cytoplasm	Nucleus	Cytoplasm	Nucleus
*noncancerous*	11 +/- 1	7.6 +/- 0.3	2.1 +/- 0.2	0.31 +/- 0.03
*immortal*	8.3 +/- 0.6	8.2 +/- 0.6	0.1+/- 0.01†	2.9 +/- 0.12
*tumorigenic*	9.6 +/- 0.7	9.1 +/- 0.5	2.8 +/- 0.4	1.6 +/- 0.2
*metastatic*	10 +/- 1	10 +/- 1	1.1 +/- 0.1	0.96 +/- 0.10

†This value in the table is obtained by using the free diffusion value *D*_*f*_ for MSH2-EGFP (mass—132 kDa), and not the MutSα-EGFP value calculated using its mass of 285 kDa. This was done because the value for the free diffusion coefficient for MutSα-EGFP is *D*_*f*_ = 6.4 +/- 0.5, which produces a pseudo-equilibrium constant that is slightly negative (K* = -0.15 +/- 0.01), which is unphysical. This may be an indication that assuming MSH2 is mostly in the form of MutSα in the cytoplasm of immortal cells is not quite true.

The standard error for the free diffusion coefficients are calculated as the same fractional error as the *D*_*eff*_ values from which the *D*_*f*_ values are scaled (by the factor (26.9/285)^1/3^ = 0.455). The fractional error in the pseudo-equilibrium constant values are found using the quadrature formula that is standard for a quantity that is derived from the ratio of two quantities: $K*=k_{on}^{*}/k_{off}$ = (*D*_*f*_/*D*_*eff*_)-1; for example see Eq 4.25 in Ref [47]. The standard error is then found from the fractional error.

In Table 8 we summarize the statistical significance for the pseudo-equilibrium constant, K*, for the MutSα protein complex across all cell types and regions. These values were determined by applying Student's t-test analysis to the data in Table 7.

**Table 8:** Statistical significance of results for the pseudo-equilibrium constant—K* for *MutSα*-EGFP *protein*.

Cell		noncancerous		immortal		tumorigenic		metastatic	
	region	*cyto*	*nuc*	*cyto*	*nuc*	*cyto*	*nuc*	*cyto*	*nuc*
noncan	*cyto*		***	***	***	ns	ns	***	***
	*nuc*			***	***	***	***	***	***
immort	*cyto*				***	***	***	***	***
	*nuc*					ns	***	***	***
tumor	*cyto*						**	***	***
	*nuc*							***	**
meta	*cyto*								ns
	*nuc*								

**Fig 4:**
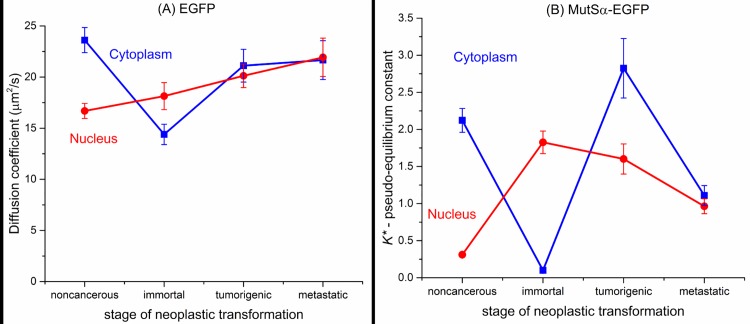
Diffusion and binding data. (A) Line plot of EGFP diffusion vs. cancer cell type (neoplastic transformation). (B) Line plot of the pseudo-equilibrium constant K* for MutSα-EGFP as a function of cancer cell type (neoplastic transformation) for both cell regions. Each x-axis is ordered according to the neoplastic progression of the cancer cell–noncancerous on the left and metastatic on the right. For each cell type cytoplasmic and nuclear values are plotted on the same graph (cytoplasm–blue; nucleus–red). Error bars are +/- SEM.

The relevant data for the discussion in the next section is summarized in line plots in Fig 4, where we plot the EGFP diffusion in Fig 4A for both cell regions and we plot the pseudo-equilibrium constant K* determined for MutSα-EGFP in the cytoplasm and the nucleus in Fig 4B.

## Discussion

A summary of the diffusion measurements is given in Fig 3. For each data point, between 18 and 55 measurements were taken–see Table 3 (mean = 31.4) for a total N = 493. In the case of MutSα-EGFP, we note that these values correspond to the effective diffusion coefficient. Since the free diffusion values for MutSα-EGFP in Table 7 (labeled *D*_*f*_) are simply scaled versions of the corresponding free EGFP *D* values, there is no merit in plotting the MutSα-EGFP *D*_*f*_ values. In the case of the MSH2 protein, we will discuss the K* results for different regions and cell types, and we will assume that these values represent the bound/free ratio (BFR) of the MutSα-EGFP complex in the nucleus. This is a reasonable assumption for the nucleus since the MMR proteins bind to the DNA in the nucleus, and in cases where a protein's binding partner is relatively immobile (like DNA for MutSα) then the pseudo-equilibrium constant K* can be physically interpreted as the bound/free ratio of MutSα (see the discussion in Sprague, et al. [48]). Since the assumption that K* is the same as the BFR requires the binding partner to be nearly fixed, we will not make this assumption in the cytoplasm, where we do not know the binding partner. Several interesting observations can be made from these data. We summarize the main results in two separate tables (Tables 9 & 10), along with a brief ‘significance and context’ statement. Additional details are given in the rest of the section that follows the two tables.

**Table 9:** Summary of *EGFP* results and their significance.

No.	Result/Observation	Significance and Context
1	Diffusion coefficients are on the order of 20 μm^2^/s in *both cytoplasmic and nuclear regions* for all cells, except immortal cells where diffusion is slower (14 μm^2^/s) in cytoplasm.	The values are in general agreement with literature values–see Supporting Information Table A in S1 Appendix. Since the size of EGFP is known, and assuming it does not bind to cellular components, the viscosity of the cytoplasm and nucleus can be determined via the Stokes-Sutherland-Einstein relation (Eq 6).
2	Monotonically increasing D values in nucleus (17–22 μm^2^/s) with increasing neoplastic transformation (normal, immortal, tumorigenic, metastatic).	Nuclear structure may be breaking down as neoplastic stages advance, causing an increase in pore size and/or reducing viscosity
3	D values are about *30% higher in cytoplasm than in nucleus for noncancerous cells*	Nucleus is more densely packed, with smaller pore sizes, than cytoplasm, thus lowering mobility in the nucleus.
4	D values are about *20% lower in cytoplasm than in nucleus for immortal cells*	The drop in D for cytoplasm for immortal cells (while the nuclear D slightly increased) is opposite what one would expect for normal cytoplasmic vs. nuclear (more dense) structures. This is possibly caused by a change in cytoplasmic properties, such as an increase in cytoplasm stiffness–see poroelastic model discussion in Moeendarbary [2]
5	D values are *36% lower in the cytoplasm of immortal cells* than in other cells	According to the poroelastic model a significant increase in cytoplasm stiffness causes a decrease in mobility–see poroelastic model discussion in Moeendarbary [2]

**Table 10:** Summary of *MutSα*­results and their significance.

No.	Result/Observation	Significance and Context
1	In the nucleus, the BFR is significantly less than one *only for the noncancerous cells* (K* = BFR = 0.31)	For noncancerous cells the need for mismatch repairs is present but small.
2	In the nuclei of all cancer cell types, the BFR equals or exceeds 1 (BFR = K*)	Cancer cells are genetically unstable & the data shows a significantly heightened repair response in terms of the number of bound MMR proteins as a result
3	In the nuclei of cancer cells, the first cancer stage has the highest bound mismatch repair (MMR) fraction (6x that of noncancerous cells) compared to subsequent cancer stages (5x & 3x over noncancerous cells respectively)	When genetic instabilities first arise during cancer initiation, the response of the MMR apparatus is at its peak as it attempts to reverse or stop the new effects; subsequent stages of cancer demonstrate that the cancer cell is increasingly better able to overcome the cell's anti-cancer response
4	In cytoplasm the most obvious change occurs for immortal cells (the first stage of cancer) where the K* is negligible (~ 0)	The response in the nucleus is so significant that it is causing nearly all MSH2 that was bound to other substrates (other proteins, perhaps cytoskeletal elements) to enter the nucleus to help repair mutating DNA

### EGFP Diffusion Results

GFP diffusion has been measured in many cells by others, but not in the cells observed here. A survey of measurements in other cells is provided in Table A in S1 Appendix. The EGFP diffusion results in Figs 3A and 4A and Table 3 indicate that the behaviors in the cytoplasm and nucleus differ. For the cytoplasm, Fig 4A clearly shows that there is a statistically significant drop (p < 0.001) by 42% in going from noncancerous to immortal cells (the first stage of neoplastic transformation). In the next stage, going from immortal to tumorigenic, the diffusion coefficient increases significantly (p < 0.001)–by 50%. In the most advanced transition stage (tumorigenic to metastatic) there is a very slight increase (~ 5%). The cause of the reduced diffusion of EGFP is probably an increase in the cytoplasmic viscosity, possibly as a result of increased crowding in the cytoplasm. Recently there have been advances in our understanding of how diffusion changes in cells are related to mechanical changes [2]. This poroelastic model is one where the filaments in cells provide structural scaffolding that is elastic, and this structure is surrounded by a viscous plasma of proteins, enzymes, organelles, and other factors and molecules. It is interesting to note that the poroelastic model and the corresponding observations by Moeendarbary et al. [2] argue that diffusion drops when stiffness increases. Thus the significant drop in diffusion in the noncancerous to immortal transition could be related to an increase in the structural stiffness in the interior of the immortal cells. One of the main characteristics of the immortal cells is the expression of h-TERT, a telomere reverse transcriptase that counteracts the normal shortening of telomeres in cell replication. Studies of mechanical properties in chronic myelogenous leukemia cells from bone marrow, using one cell line with hTERT and another with hTERT knockdown, indicated significant changes in the cytoskeleton with hTERT. Using the method of micropipette aspiration to measure the viscoelasticity of cells, Zhang et al. [51] found that the hTERT knockdown cells (KAT cells) had higher elastic moduli than the cells with hTERT (K562 cells), indicating that the KAT cells are less deformable than K562 and KC cells. However, they measured no statistically significant difference between the two cell types for the viscous modulus. The poroelastic model suggests stiffer cells result in decreased diffusion, and the fact that KAT cells (hTERT knockdown) are stiffer than K562 cells (hTERT present) suggests that hTERT cells should exhibit increased diffusion, which is opposite what we observe in going from noncancerous (no hTERT) to immortal cells (hTERT present). However, the two cell types differ (bone marrow vs. mammary epithelial cells), and so they behave differently. In our group's previously reported AFM stiffness measurements on the same mammary cells [5], the stiffness increased when hTERT was present (for immortal cells), and our corresponding decrease in diffusion agrees with the prediction of the poroelastic model. In both cell type cases, the results suggest that the presence of hTERT changes the cytoskeletal structure, which could contribute to our observations for immortal cells.

Another mechanism that could explain the observed changes in the cytoplasmic D values of EGFP in the different cell lines is cytoskeletal reorganization. Previous studies have implicated the p53 gene in cytoskeletal reorganization by interaction through intermediate filaments and actin [52,53]. Other studies have suggested Ras and Ras-related GTPases exhibit some control over actin reorganization [54,55]. This cytoskeletal reorganization affects the macroscopic properties of the whole cell [3,4,56]; such reorganization could be the cause of the changes we have observed in diffusion coefficients as a function of the stage of neoplastic transformation. A careful consideration of cellular reorganization would include the contribution of obstacles, microdomains, as well as nuclear and cytoplasmic structures. All of these could act as sources for variation in the diffusion coefficient. To confirm that diffusion might not always be solely driven by Brownian motion, and thus potentially explain the origin of the different diffusion behaviors observed in the various cell stages and various cellular regions, one could extend our results by performing FRAP measurements using bleaching spots of various sizes, and then plot the diffusion time as a function of the bleaching radius, e.g. see the work by Lenne, et al. [57]. Another good example where the spot size is varied or different bleaching patterns are used is the recent work of Pincet et al.[58]. Using FRAP, they were able to differentiate between the Brownian motion of free diffusion and combinations of diffusion with restricted motion in the behavior of lipids in three different membrane platforms.

In the nucleus, EGFP mobility increases monotonically with increasingly severe neoplastic transformation (Fig 4A). Since EGFP is not known to specifically bind to any location in the nucleus, the faster diffusion rates could be due to the rearrangement of the nuclear matrix to create lower viscosities as the cells advance in neoplastic stages. A previous study of nuclear proteins in breast cancer cells implicated the protein P114 to strongly bind DNA fragments to matrix attachment regions within the nucleus [59]. The P114 binding activity was observed in human breast carcinomas but not in normal or benign breast cells. Another study showed the protein P300 induced nuclear morphological changes that correlated to cancer prognosis in prostate cells [60]. P300 induces histone acetylation, which changes the chromatin structure. The authors also suggest that expression of P300 is mediated by lamin A and lamin C, which are the constituent components of the nuclear matrix. Thus, there are studies that have reported nuclear reorganization as a result of cancer, and such reorganization could explain our observations of monotonically increasing D values in the nucleus for EGFP. Another possible explanation for the monotonic increase in nuclear D for EGFP is, according to a poroelastic model, the stiffness of elastic structures in the nucleus is decreasing modestly under neoplastic transformation. The mechanism could also be a combination of the two effects, nuclear reorganization or a change in elastic stiffness of nuclear structures.

### MutSα Diffusion and Binding Ratio Results

Here we discuss the MutSα-EGFP results for diffusion and the pseudo-equilibrium constant (bound/free ratio). First we point out that the one-dimensional diffusion of MutS proteins, once they bind to DNA to scan for, or repair, DNA mismatches has been measured by Cho et al. [61] in a set of ingenious measurements using single molecule microscopy that followed the diffusion (and rotation) of MutS as it translated and rotated along a 15 kbase section of DNA that was straightened in a flowing experiment. From observations yielding the mean-squared-displacement vs. time of MutS, they found that the MutS diffusion coefficient while searching for errors was *D*_MutS,searching_ = 0.035 ± 0.005 μm^2^/s (mean ± s.e.); and when MutS was bound to a mismatch—*D*_MutS,mismatch_ ≤ 0.002 ± 0.002 μm^2^/s (mean ± s.e). This means that when MutS is bound to DNA we can ignore the size of its diffusion compared to the values we measured for the effective diffusion of the same protein (*D*_*eff*_ ~ 3–7 μm^2^/s), regardless of cell type.

Here we discuss the MutSα-EGFP results for diffusion and the pseudo-equilibrium constant (bound/free ratio), which was summarized for both cell regions and all cell types in Table 7 and plotted for both cell regions in Fig 4B. The most likely binding partner in the nucleus is DNA since MutSα is a mismatch repair protein complex. In the cytoplasm, the binding partner is not known. However, numerous MSH2 binding partners are known, two of which were discussed earlier. In fact, recently Chen et al. [62] found that the protein WDHD1 stably binds to both MutSα and MutSβ complexes.

For the nuclear region, the MutSα pseudo-equilibrium constant or bound/free ratio (BFR) values in Table 7 and Fig 4B tell an interesting story. The most obvious observation is that the BFR value for noncancerous cells is significantly lower (3-6x lower) than the three cancer cell types. For every MutSα-EGFP complex bound to DNA in the nucleus of the noncancerous cells there are three unbound ones. The biggest change in this ratio occurs in the transition to the first stage of cancer, where for the immortal cells there are close to two (1.83) MutSα-EGFP complexes bound to DNA for every one that is free, a substantial reversal from the noncancerous cells. This result is important since it demonstrates the cells response to the genetic insults caused by cancer at the very beginning of the transformation. In the next stage (tumorigenic), the number of bound MutS complexes drops a bit to 1.6 for every free complex, indicating perhaps that at this stage, the MMR proteins are being inactivated to a better extent than they were at earlier stages. This same trend is exhibited in the last cell type, where the metastatic BFR value has dropped to roughly equal numbers of bound and free MMR protein complexes, further demonstrating the deteriorating ability of the MMR apparatus to keep up with the ever increasing genetic instability induced by the most advanced stages of cancer.

For the cytoplasmic region, the MutSα K* values in Table 7 and Fig 4B tell another interesting story. The most obvious observation is that the K* value for immortal cells is extremely low–very close to zero. This shows that nearly all MutSα complexes are free to move from the cytoplasm into the nucleus to assist with repairing the increased number of insertions/deletions and single nucleotide polymorphisms in the early stages of cancer onset. In the next two cancer stages (tumorigenic and metastatic) the cytoplasmic K* values are back up substantially to values that exceed those in the nucleus. Perhaps this indicates the ability of late cancer stages to reduce nuclear transport of MutSα. The noncancerous cells have a rather high cytoplasmic K*, perhaps indicating a much reduced need for mismatch repairs in the nucleus than for cancer cells. The K* peak occurs for tumorigenic cells (2.83), and then the value declines to one for metastatic cells, which matches the value in the nucleus. Our data agrees with observations by Christmann, et al. [32] for HeLa MR cells that were further genetically compromised by the alkylating agent MNNG. In their experiments they found that nuclear levels of MSH2 increased 6-fold fairly linearly with time, after exposure to the alkylating agent. The increased nuclear MSH2 was shown to come from the cytoplasm, not via transcription. Before the alkylating agent was introduced, the cytoplasm had 5x as much MSH2 as the nucleus, and 2 hours after exposure to the alkylating agent, the ratio had flipped with 6x as much MSH2 in the nucleus as in the cytoplasm.

Another interesting study of MSH2 behavior in cells exposed to the same MNNG alkylating treatment used an MSH2-null human endometrial cancer cell line (Hec59) and then investigated behavior of a set of MSH2 mutants, along with wild-type MSH2 as a control [63]. They found that, after treating MSH2(WT), MSH2(D167H mutant) and MSH2(K393M mutant) cells with MNNG, a MMR complex containing MLH1, MSH2, MSH6, and PCNA formed. However MMR protein complex formation was not detected in the MSH2(R524P mutant) cell line, and they discussed that this was consistent with the inability of this mutant to localize to chromatin. The fourth mutant—MSH2(P622L)–did not interact with MLH1 and PCNA, which they concluded was most likely due to a defect in protein stability and/or heterodimer formation with MSH6 that affects chromatin localization. Both this MSH2 study, and the one mentioned above by Christmann, et al. [32], indicate that MSH2 bound to MSH6 plays a very large role in repair of damaged DNA due to alkylating agents.

A metric that quantifies the cancer response of the MMR protein apparatus for each distinct cell type is the ratio of K* values of the nucleus and the cytoplasm. Physically this is a sort of measure of how actively the cytoplasm and nucleus jointly respond to the threat of genetic instability posed by cancer. This ratio of K* values will be called the Cancer Response Activity (CRA) and is mathematically defined as

$$CRA=\frac{K{*}_{nuclear}}{K{*}_{cytoplasm}}.$$ (11)

**Fig 5:**
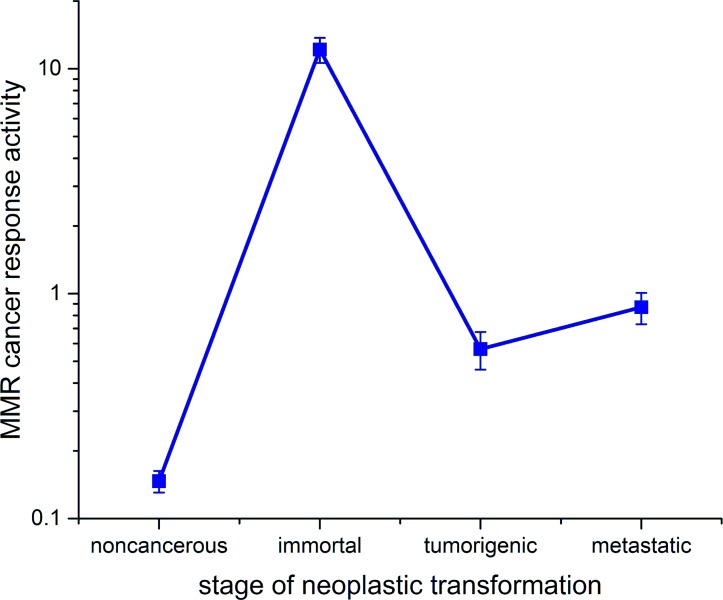
Log_10_ scale plot of the cancer response activity (CRA) vs. cancer cell type (stage of neoplastic transformation).

A plot of CRA vs cell type is given in Fig 5. This plot shows a minimum for noncancerous cells, as expected, and a dramatic peak for immortal cells that is on average 20x the value of the CRA for the other 3 cell types, and close to 100 x higher than that for noncancerous cells. The clear trend is that the CRA declines as cancer progresses toward the most advanced stage of metastasis.

Finally, the CRA metric could be valuable as a cancer diagnostic–it is most sensitive at early onset, which is exactly when you want to be able to detect the seeds of cancer.

## Conclusions

In this study we have used FRAP to measure the diffusion of free EGFP and of MSH2-EGFP, which we have assumed is bound to MSH6 to form MutSα in both the cytoplasm and nucleus of four different cell types. The cells were chosen to represent, to some degree, the different stages of neoplastic transformation, namely noncancerous, immortal, tumorigenic, and metastatic.

The diffusion data on free EGFP indicates there are two main results: 1) diffusion in the cytoplasm is significantly lower for immortal cells than for all other cell types and 2) diffusion in the nucleus monotonically rises as we undergo neoplastic transformation from noncancerous cells to metastatic cells. We interpret these to mean that the cancer response in the first stage of cancer (immortal cells) raises the viscosity of the cytoplasm and that the viscosity of the nucleus may be dropping as cancer progresses. The second observation may also be indicative of structural remodeling occurring in the nucleus, for which there is significant evidence in the literature [64].

The nuclear data on MutSα- EGFP shows that the pseudo-equilibrium constant for noncancerous cells is significantly lower (3-6x lower) than for the three cancer cell types. Since the binding partner for MutSα is known to be DNA here, we can conclude that K* corresponds to the bound-free ratio so that the nuclear BFR is significantly higher in cancer cells than in noncancerous ones, as one might expect. However the largest increase occurs for immortal cells, indicating that the response is greatest in this first stage of neoplastic transformation than in subsequent stages, a result that mirrors the EGFP cytoplasm results. In addition, we saw that the K* value for cytoplasmic MutSα- EGFP is nearly zero for immortal cells, and stays fairly high for all the other cells, further evidence for the unique changes underway in the first stage of neoplastic transformation.

Finally, we have also proposed a metric for measuring cancer response activity as the ratio of nuclear to cytoplasmic K* values that jumps a factor of 100 in going from noncancerous to cancerous cells during the earliest stage of cancer. Such a metric might be helpful in identifying the onset of cancer.

## Supporting Information

S1 Appendix Diffusion of EGFP in other cells. This Appendix provides values from the literature for diffusion coefficients measured for free EGFP in various cells. **Table A: EGFP Diffusion Coefficients.** EGFP diffusion coefficients measured in various cells (from the literature).
(DOCX)
