# Supplementary material for: Diffusion and Binding of Mismatch Repair Protein, MSH2, in Breast Cancer Cells at Different Stages of Neoplastic Transformation
Source: PLoS One. 2017 Jan 26;12(1):e0170414. doi: 10.1371/journal.pone.0170414 (PMC5268495; doi:10.1371/journal.pone.0170414)
Supplement: S1 Appendix — This Appendix provides values from the literature for diffusion coefficients measured for free EGFP in various cells. Table A: EGFP Diffusion Coefficients. EGFP diffusion coefficients measured in various cells (from the literature). (DOCX) [file pone.0170414.s001.docx]

Supporting Information

## **S1 Appendix: Diffusion of EGFP in other cells**

Other researchers who have measured diffusion coefficients of free EGFP have reported a range of values between 20 um^2^/s and 54 um^2^/s. S1 Table is a list of measured EGFP diffusion coefficients.

**Table A. EGFP diffusion coefficients measured in various cells (from the literature).**

| **Cell** | **D (um^2^/s)** | **Method** | **Reference** |
| --- | --- | --- | --- |
| COS7 | 41.3 +/- 8.2 | FRAP | M. Kang, et al. [1] |
| COS7 | 43.6 | FRAP | M. Kang, et al. [2] |
| COS7 | 35 (cyto); 23 (nucleus) | FRAP | M. Kang, et al., [3] |
| COS7 | 45 (cytoplasm) | FCS | Goodwin, et al., [4] |
| HEK293 | 54 +/- 12 (monomeric) 34 (dimers) | FCS | N. Dross, et al. [5] |
| HELA | 53 +/- 12 (monomeric) 30 (dimers) | FCS | N. Dross, et al. [5] |
| TP366 | 51 +/- 12 (monomeric) 32 (dimers) | FCS | N. Dross, et al. [5] |
| T98G | 51 +/- 12 (monomeric) 31 (dimers) | FCS | N. Dross, et al. [5] |
| NIH3T3 euchromatin | 1. /- 1.5 | FCS | A. Bancaud, et al. [6] |
| CHO XrS6 | 29.3 +/- 3.7 (cyto); 27.5 +/- 4.2 (nucleus) | FCS | Dennis Merkle, et al. [7] |
| CHO | 25-30 (cyto) | FRAP | Dayel, et al. [8] |
| CHO-K1 | None reported | FRAP | Swaminathan, et al. [9] |
| PTK2 | 21+/- 8 (cyto) 20±7 (nucleus) | FCS | Z. Wang, et al. [10] |
| Dictyostelium | 20 +/- 4 (cyto) | FCS | Ruchira, et al. [11] |
| HeLa | 25.1 +/- 1.2 (cyto); 23.0 +/- 1.0 (nucleus) | scFCS | G. Maertens, et al. [12] |
| HeLa | 33.3 +/- 3.6 (nucleus) | FRAP | Braga, et al. [13] |
| HeLa | 25.2+/-4.5 (cyto) 23.5+/-5.3 (nucleus) | FCS | Chen, et al. [14] |
| HeLa | 27.57 +/- 4.4 (cyto) | FCS | Guo et al. [15] |

## **Supporting Information References**

1. Kang M, Day CA, Kenworthy AK, DiBenedetto E. Simplified equation to extract diffusion coefficients from confocal FRAP data. Traffic Cph Den. 2012;13: 1589–1600. doi:10.1111/tra.12008

2. Kang M, Day CA, DiBenedetto E, Kenworthy AK. A quantitative approach to analyze binding diffusion kinetics by confocal FRAP. Biophys J. 2010;99: 2737–2747. doi:10.1016/j.bpj.2010.09.013

3. Kang M, Day CA, Drake K, Kenworthy AK, DiBenedetto E. A generalization of theory for two-dimensional fluorescence recovery after photobleaching applicable to confocal laser scanning microscopes. Biophys J. 2009;97: 1501–1511. doi:10.1016/j.bpj.2009.06.017

4. Goodwin JS, Drake KR, Rogers C, Wright L, Lippincott-Schwartz J, Philips MR, et al. Depalmitoylated Ras traffics to and from the Golgi complex via a nonvesicular pathway. J Cell Biol. 2005;170: 261–272. doi:10.1083/jcb.200502063

5. Dross N, Spriet C, Zwerger M, Müller G, Waldeck W, Langowski J. Mapping eGFP Oligomer Mobility in Living Cell Nuclei. Rappoport JZ, editor. PLoS ONE. 2009;4: e5041. doi:10.1371/journal.pone.0005041

6. Bancaud A, Huet S, Daigle N, Mozziconacci J, Beaudouin J, Ellenberg J. Molecular crowding affects diffusion and binding of nuclear proteins in heterochromatin and reveals the fractal organization of chromatin. EMBO J. 2009;28: 3785–3798. doi:10.1038/emboj.2009.340

7. Merkle D, Zheng D, Ohrt T, Crell K, Schwille P. Cellular dynamics of Ku: characterization and purification of Ku-eGFP. Chembiochem Eur J Chem Biol. 2008;9: 1251–1259. doi:10.1002/cbic.200700750

8. Dayel MJ, Hom EF, Verkman AS. Diffusion of green fluorescent protein in the aqueous-phase lumen of endoplasmic reticulum. Biophys J. 1999;76: 2843–2851. doi:10.1016/S0006-3495(99)77438-2

9. Swaminathan R, Hoang CP, Verkman AS. Photobleaching recovery and anisotropy decay of green fluorescent protein GFP-S65T in solution and cells: cytoplasmic viscosity probed by green fluorescent protein translational and rotational diffusion. Biophys J. 1997;72: 1900–1907. doi:10.1016/S0006-3495(97)78835-0

10. Wang Z, Shah JV, Chen Z, Sun C-H, Berns MW. Fluorescence correlation spectroscopy investigation of a GFP mutant-enhanced cyan fluorescent protein and its tubulin fusion in living cells with two-photon excitation. J Biomed Opt. 2004;9: 395–403. doi:10.1117/1.1646416

11. Ruchira, Hink MA, Bosgraaf L, van Haastert PJM, Visser AJWG. Pleckstrin homology domain diffusion in Dictyostelium cytoplasm studied using fluorescence correlation spectroscopy. J Biol Chem. 2004;279: 10013–10019. doi:10.1074/jbc.M310039200

12. Maertens G, Vercammen J, Debyser Z, Engelborghs Y. Measuring protein-protein interactions inside living cells using single color fluorescence correlation spectroscopy. Application to human immunodeficiency virus type 1 integrase and LEDGF/p75. FASEB J Off Publ Fed Am Soc Exp Biol. 2005;19: 1039–1041. doi:10.1096/fj.04-3373fje

13. Braga J, Desterro JMP, Carmo-Fonseca M. Intracellular macromolecular mobility measured by fluorescence recovery after photobleaching with confocal laser scanning microscopes. Mol Biol Cell. 2004;15: 4749–4760. doi:10.1091/mbc.E04-06-0496

14. Chen Y, Müller JD, Ruan Q, Gratton E. Molecular brightness characterization of EGFP in vivo by fluorescence fluctuation spectroscopy. Biophys J. 2002;82: 133–144. doi:10.1016/S0006-3495(02)75380-0

15. Guo M, Ehrlicher AJ, Jensen MH, Renz M, Moore JR, Goldman RD, et al. Probing the Stochastic, Motor-Driven Properties of the Cytoplasm Using Force Spectrum Microscopy. Cell. 2014;158: 822–832. doi:10.1016/j.cell.2014.06.051
